# Polysaccharides from *Asparagus officinalis* L.: phytochemistry, health benefits, biomedical, and food industry applications

**DOI:** 10.3389/fphar.2026.1842554

**Published:** 2026-06-03

**Authors:** Lihong Wu, Haizheng Bi, Mengru Zhang, Xinrui Xu, Meng Wang

**Affiliations:** Key Laboratory of Basic and Application Research of Beiyao Ministry of Education, Heilongjiang University of Chinese Medicine, Harbin, China

**Keywords:** applications, *Asparagus officinalis* L., health benefits, plant chemistry, polysaccharides

## Abstract

Asparagus officinalis L. (*A. officinalis*) is a highly representative plant in the *Asparagus* L. genus, with both edible and medicinal values. As a typical macromolecular metabolite in *A. officinalis*, *A. officinalis* L. polysaccharides (AOPs) have attracted much attention due to its rich functional biopolymer properties. AOPs have been proven to have cytotoxic and pro-apoptotic effects, immune regulation, and regulation of intestinal flora, anti-neuritis, anti-atherosclerosis, anti-non-alcoholic liver disease, and other health benefits. Among them, regulation of intestinal flora is the most important health benefit of AOPs. In addition, researchers have developed corresponding drug formulations based on their anti-inflammatory, cytotoxic, and pro-apoptotic effects in practical applications. Through a review of previous studies, researchers have found that plant residues extracted from *A. officinalis* can be developed into high-value cellulose nanocrystals. This also provides a valuable reference for the reuse of other natural plant waste. In this study, we summarize and review research on AOPs’ phytochemical structures, biomedical, and industrial applications, based on the latest research findings and practical considerations.

## 1 Introduction

**FIGURE 1:**
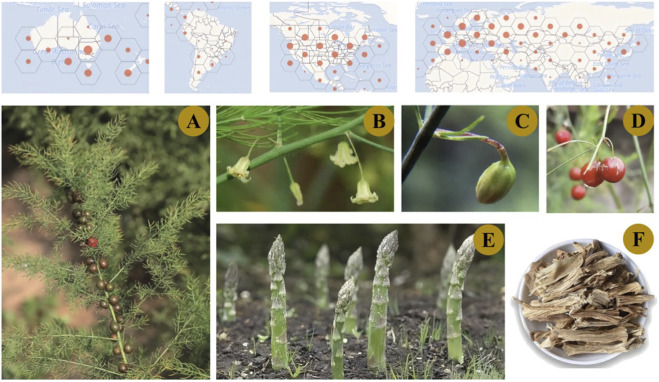
Plant characteristics and geographical distribution of *Asparagus officinalis* L. **(A)** Whole plant; **(B)** flowers; **(C)** ovaries; **(D)** fruit; **(E)** spear; **(F)** dried medicinal herbs (Source: https://www.gbif.org. For an intuitive introduction to the plant morphology, the images are processed and quoted to show the characteristics of different parts from network and public sources).

*Asparagus officinalis* L. (*A. officinalis*) is a highly representative plant of the *Asparagus* L. genus. *A. officinalis*, traditionally, has its seedlings as the main edible and medicinal parts, and it has also been the main research object of researchers in recent years (Figure 1). In ancient Greece and Rome, *A. officinalis* was already used to treat gout. In traditional Chinese medicine theory, *A. officinalis* is believed to promote excretion and enhance immunity, making it an important metabolite in dietary therapy (Xia et al., 2025). In modern times, *A. officinalis* is regarded as a plant with great nutritional and research value due to its high content of dietary fiber, vitamins, minerals, and plant metabolites and is highly favored by consumers and researchers (Sun L. et al., 2025; Zhu et al., 2025). The nutritional composition analysis of *A. officinalis* is shown in Table 1. *A. officinalis* polysaccharides (AOPs), a natural heteropolysaccharide derived from renewable resources, have been proven by researchers to have extremely low toxicity and immunogenicity (Czapalay et al., 2025). It exhibits excellent biodegradability and biocompatibility. AOPs also demonstrate great potential in biotechnology and nanoformulation. According to the relevant regulations of international regulatory agencies such as the US Food and Drug Administration and the European Food Safety Agency, AOPs with the above characteristics are considered safe and in line with the research concepts of the new era.

**TABLE 1:** Nutrients of *Asparagus officinalis* L. (per 100 g) (source: https://nlc.chinanutri.cn).

Nutritional type	Project	Content	Ranking among similar plants	Mean of the same category
Energy and related metabolites	Edible	90%	25	85%
	Water	93.00 g	27	86.8 g
	Energy	111.00 kJ	31	204 kJ
	Protein	1.40 g	54	3.2 g
	Fat	0.10 g	48	0.4 g
	Ash	0.60 g	54	1.3 g
	Carbohydrate	4.90 g	18	8.3 g
Vitamin	Carotene	100.00 μg	43	1,061.6 μg
	Thiamin	0.04 mg	21	0.04 mg
	Riboflavin	0.05 mg	47	0.09 mg
	Niacin	0.70 mg	17	0.62 mg
	Vitamin C	45.00 mg	11	25.0 mg
Mineral	Ca	10.00 mg	61	84 mg
	P	42.00 mg	32	56 mg
	K	213.00 mg	32	254 mg
	Na	3.10 mg	58	66.0 mg
	Mg	10.00 mg	57	32 mg
	Fe	1.40 mg	33	2.4 mg
	Zn	0.41 mg	34	0.74 mg
	Se	0.20 μg	51	1.07 μg
	Cu	0.07 mg	34	0.17 mg
	Mn	0.17 mg	48	0.54 mg

**FIGURE 2:**
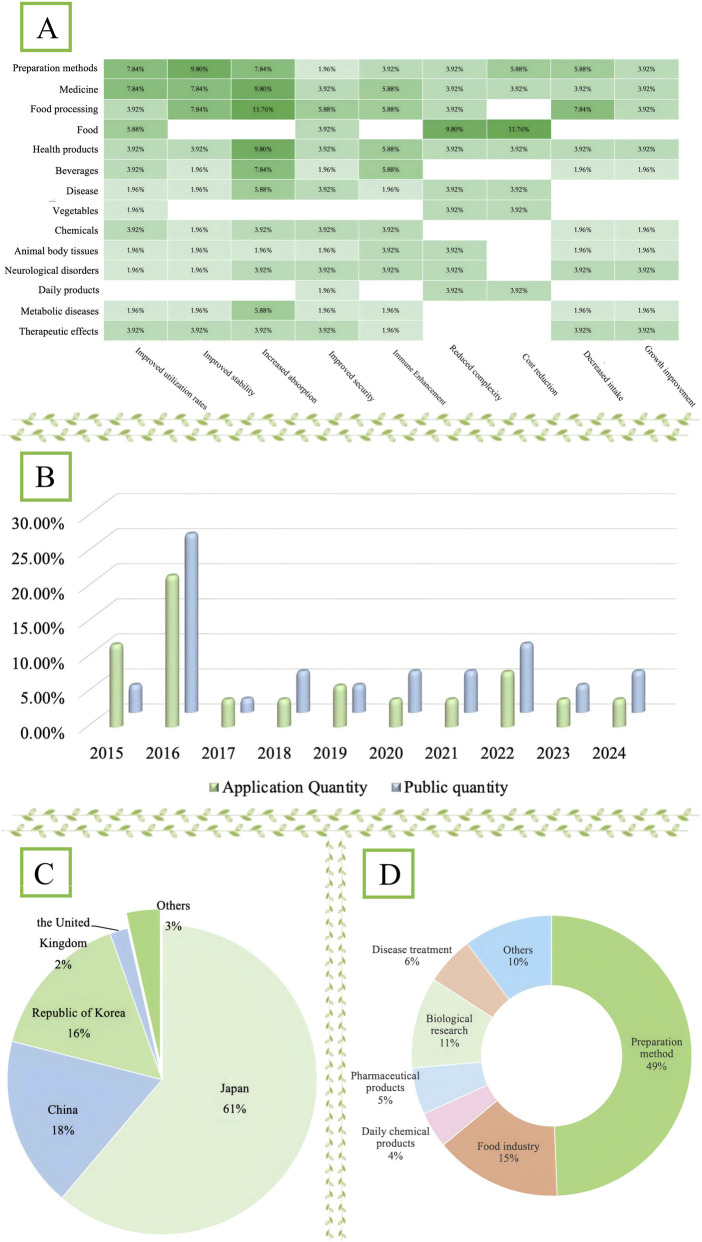
**(A)** The technical efficacy value of patents in various fields. **(B)** Patent application trends and disclosure trends. **(C)** Patent application status in various countries. **(D)** Patent application status in various fields.

Researchers have successfully extracted typical non-starch AOPs, such as pectin and cellulose, from non-traditional edible parts such as *A. officinalis* stems and roots (Rajakaruna et al., 2025; Zhang J. et al., 2025). In studying *A. officinalis* pectin polysaccharides, researchers mainly focus on their unique pectin arabinose galactan complex structure (Shahin et al., 2025). In studies of cellulose polysaccharides, researchers focus on developing its nanocrystals. As a traditional medicinal and edible plant, *A. officinalis* has fully demonstrated its development value in the food industry and pharmaceutical preparations (Chen et al., 2024; Li et al., 2024). However, through statistical analysis of the number of patent applications and publications in recent years, it is not difficult to observe that AOPs have also achieved good results in practical applications. AOPs, as a representative class of macromolecular polymers in *A. officinalis*, have been introduced in various fields, such as food, daily chemical products, and pharmaceuticals (Zhu et al., 2024). Regarding food, AOPs are mostly used as an additive in beverages. In daily chemical products, AOPs are also added to lotion. In terms of drug development, researchers mainly focus on their anti-inflammatory and anti-cancer activities. At present, the published patents for AOPs mainly focus on three aspects: innovative extraction methods, food research and development, and drug development (Komatsu and Matsunaga, 2024; Zhu et al., 2024). According to the 2015–2024 data report, since the significant increase in applications and publications in 2016, the overall number has shown a relatively stable state. The main development and patent applications of AOPs are shown in Figure 2.

Research so far has confirmed that AOPs have pharmacological activities, such as cytotoxic and pro-apoptotic effects, immune regulation, regulation of intestinal flora, anti-neuritis, anti-atherosclerosis, and anti-nonalcoholic liver disease. Among them, treatment of non-alcoholic liver disease and atherosclerosis mainly depends on the regulation of intestinal flora. Therefore, regulating gut microbiota is the most important health benefit of AOPs (He et al., 2024; Yang et al., 2024). In traditional medicine, *A. officinalis* is considered to have anti-oxidant, anti-inflammatory, and anti-bacterial effects, among which its anti-oxidant capacity is 1.8 times that of tomatoes. Previous studies have suggested that the high content of asparagine in *A. officinalis* is the main reason for its outstanding anti-oxidant capacity (Ma et al., 2024). However, recent research results have shown that the synergistic effect of AOPs also plays a significant positive role. Compared with other small-molecule metabolites in *A. officinalis*, the chemical structure of AOPs is complex, with a block-like spatial conformation and stronger signal activation ability. In terms of pharmacological activity, *A. officinalis* has multi-target and low-toxicity characteristics (Javaid et al., 2024; Xie et al., 2024). At the same time, *A. officinalis* is rich in dietary fiber polysaccharides, which are superior to the single mechanism of small molecules in terms of mechanism of action. As mentioned above, AOPs have significant advantages in terms of safety, efficacy, and industrial feasibility. Therefore, the application space of AOPs is broad and worthy of expectations.

To better promote the subsequent research and development application of AOPs, we systematically evaluate their emerging potential in biomedical and pharmaceutical formulations. In addition, for the purpose of better analyzing the structure–activity relationships of AOPs, we focus on the phytochemical composition and function of AOPs while combining molecular mechanisms for in-depth analysis. In summary, this review provides a comprehensive framework to promote the translational application of AOPs in biomedical and daily chemical industries and to provide a reference for future researchers to address the technological gap.

## 2 Methodology

**FIGURE 3:**
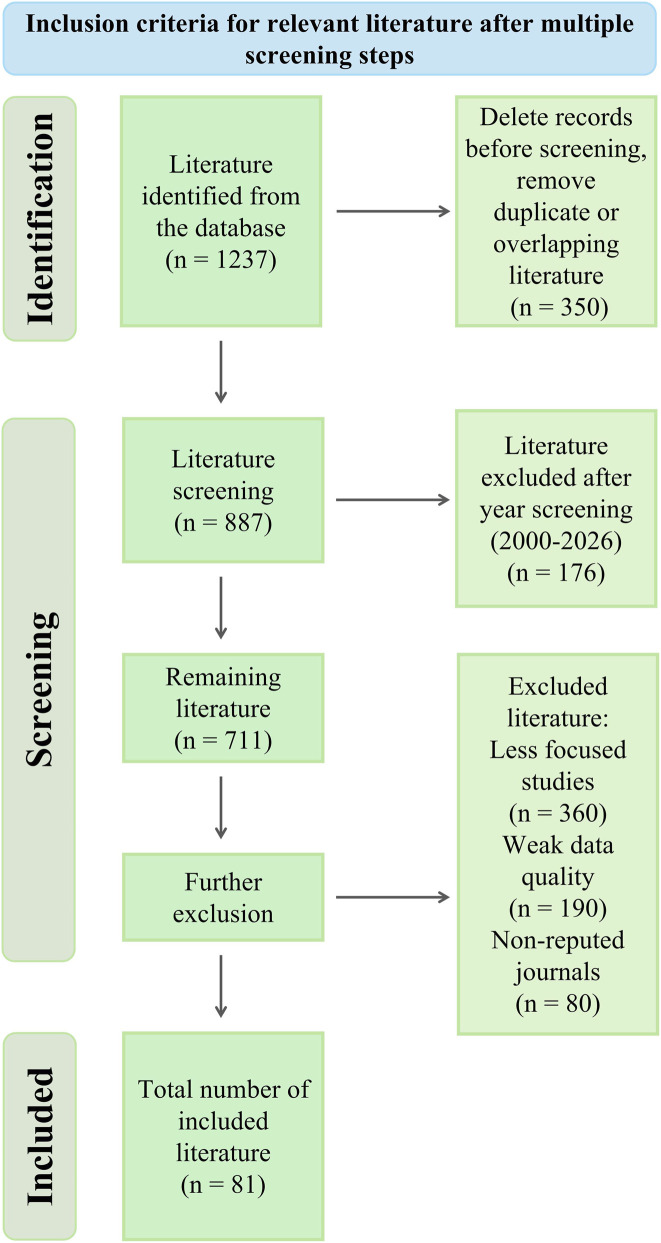
PRISMA flow diagram summarizing the screening process for selection of suitable studies.

Publications published from 2000 to 2026 were retrieved from databases such as PubMed, Google Scholar, and Web of Science. To search for the literature, this article uses keyword combinations, including *A. officinalis*, *A. officinalis* polysaccharides, Asparagus, and Asparagus polysaccharides, and further refines the selection by applying Boolean operators (Figure 3). To ensure the scientific and effective content of the article, only English publications are included as references in this article, and further screening is conducted based on the titles, abstracts, and content of the relevant literature. We excluded the repetitive literature, the literature whose research type was inconsistent with this article, and the literature whose main research object was other metabolites in *A. officinalis*. This review focuses on the plant chemical structure, biomedical applications, and industrial applications of AOPs and integrates them using a structured and systematic approach. As the focus of this article was on explaining the activity of AOPs, no meta-analysis was conducted.

## 3 Innovative exposition

Although there have been review articles on polysaccharides in the *Asparagus* L. genus, previous studies have mainly focused on food research and lacked discussions on structure–activity relationships (Chen et al., 2026). *A. officinalis*, as an important medicinal and edible homologous plant, has an important pharmacological value that cannot be ignored. At the same time, the difference in polysaccharides before and after *A. officinalis* fermentation and the cellulose nanocrystals derived from *A. officinalis* deserve special attention. Therefore, AOPs were reviewed more comprehensively and deeply in this study.

By comparing AOPs before and after fermentation with the lactic acid bacteria strain NCU116, researchers obtained valuable information. In terms of monosaccharide composition, the molar ratio of Rha and GalUA in AOPs after fermentation (F-AOPs) changed from 0.05 to 0.07 and from 2.10 to 5.19, respectively, and the content of Xyl decreased significantly so that it could not be detected. This shows that the fermentation process can significantly change the monosaccharide composition of polysaccharides. At the same time, the molecular weight of AOPs decreased from 181.3 kDa to 152.8 kDa after fermentation. However, the HPSEC chromatogram showed that the molecular weight distribution of AOPs before and after fermentation had a wide range and showed asymmetric peaks. Therefore, although it has been proven that fermentation affects the molecular weight of polysaccharides, due to the limitations of purification methods and analytical instruments, more specific conclusions have not been reached.

It is worth noting that researchers have achieved some valuable results in the rheology, particle size, and zeta potential of AOPs. Under the condition of 25 °C shear rate of 50–1,000 s^−1^, the apparent viscosity of polysaccharides before and after fermentation was in the mPa level, but the apparent viscosity of F-AOPs was higher and was positively correlated with polysaccharide concentration. It is generally believed that the increase in viscosity may be related to the restriction of the movement and stretching of polymer chains, so F-AOPs were considered to have a more complex branched chain structure. In addition, although the particle size of polysaccharides increased in a concentration-dependent manner before and after fermentation, the average particle size of 20 mg/ml F-AOPs was equivalent to that of 5 mg/ml AOPs, suggesting that fermentation had a significant impact on the zeta potential of polysaccharides. The higher concentration of nanoparticles is conducive to the formation of an emulsion with a smaller droplet size, so the nano-preparation would be more stable. It is noteworthy that even at a low concentration of 1 mg/ml, the zeta potential of AOPs before and after fermentation exceeded | ±30|. The zeta potential value was used as an index of the stability of the polysaccharide solution, which further proved that the AOP solution had good stability before and after fermentation.

At the same time, due to the botanical characteristics of *A. officinalis* rich in cellulose, after the amorphous region of cellulose is hydrolyzed through sulfuric acid hydrolysis, biodegradable cellulose nanocrystals with high purity, high crystallinity, and good biocompatibility can be obtained. At present, researchers have confirmed that cellulose nanocrystals from *A. officinalis* can act as an emulsion stabilizer in Pickering emulsions and can be used as a food-grade emulsifier for industrial production. In addition, cellulose nanocrystals from other plants have the same high biocompatibility, biodegradability, and extremely low cytotoxicity and have also made some valuable achievements in wound care and drug delivery. It mainly includes targeted drug delivery as a drug carrier and wound dressing loaded with antibacterial substances. However, the plant materials of the more mature technology are mostly from trees. To better fit the concept of green and environmental protection, many researchers have focused on plant waste in recent years, such as grape pomace and papaya stem. Since *A. officinalis* can harden quickly after harvest, only a small part can be fully utilized, and the remaining is usually used to produce low-value products. Therefore, the possibility of developing cellulose nanocrystals provides a broad prospect for the research and development of new high-value-added *A. officinalis* products.

## 4 Plant chemical functions and physicochemical properties

### 4.1 The influence of different preparation processes

The biological changes in polysaccharides can exhibit their inherent high specificity and efficiency. In previous studies, researchers have noted that different storage conditions can lead to the modification of polysaccharides in *A. officinalis* cell walls. Among them, storage temperature and time are the most significant factors, and the results of this effect are most intuitively reflected in the changes in neutral sugars and uronic acids (Redondo-Cuenca et al., 2023; Wang et al., 2022). Therefore, to better study the differences in polysaccharide structure and activity under different preparation processes, researchers fermented *A. officinalis* slurry inoculated with NCU116 and extracted polysaccharides from it.

Researchers extracted *A. officinalis* powder and fermented it in a ratio of 1:20 using deionized water. According to the analysis results of high-performance anion exchange chromatography (HPAEC), researchers observed significant changes in the monosaccharide composition of polysaccharides in *A. officinalis* slurry after fermentation. Its main manifestation is that glucuronic acid (GlcUA) can be detected, while xylose (Xyl) cannot be detected, and the content of uronic acid is also significantly increased. This phenomenon is believed to be caused by microbial enzymes’ degradation of cell wall pectin, the synthesis of microorganisms themselves, and the acidic environment resulting from fermentation (Wang et al., 2021). In subsequent studies, researchers chose to extract AOPs in 0.1 M HCl (Zhu et al., 2024). They set the liquid–solid ratio to 1:15 to better demonstrate the effect of acidity and alkalinity on the extraction process. However, there are significant differences in monosaccharide composition, and in comparison, different extraction methods can significantly impact monosaccharide composition.

Under normal conditions, researchers mainly use hot water extraction (HWE) and ultrasonic circulation extraction (UCE) to extract AOPs (Table 2). The polysaccharide extraction rate obtained by researchers through the HWE method ranges from 4.19% to 9.5% (Zhao et al., 2012). This extraction method is simple and inexpensive, so it often appears in large-scale extraction in laboratories and factories. However, HWE also has drawbacks, such as excessively long extraction times. Therefore, to better adapt to industry demands, researchers have further developed UCE technology. Researchers optimized the extraction process parameters using three factors and three levels, with ultrasound power, extraction time, and liquid–solid ratio as the main factors. The final optimal extraction conditions were ultrasonic power of 600 W, extraction time of 46 min, and a liquid–solid ratio of 35 ml/g. Under these conditions, the polysaccharide yield was 3.134%. This method has a shorter extraction time, better ultrasonic cavitation effect, and does not affect the extraction rate. It is currently the ideal extraction method.

**TABLE 2:** Extraction methods and polysaccharide composition of *Asparagus officinalis* L. polysaccharides.

Extraction methods	Name	Time	Temperature	Solid–liquid ratio	Yield	Molecular weight	Monosaccharide composition	Structure	Ref
HWE	AP	2 h	100 °C	1:30 w/v	4.19%	8.457, 4.001 and 2.612 kDa	Rha: Ara: GlcN: Gal: Glc: GlcNAc: Xyl: Man: Fru: Rib = 1.4%: 5.9%: 0.8%: 1.7%: 36.9%: 3.7%: 1.8%: 2.9%: 42%: 2.8%	3,600–3,200 cm^−1^: a characteristic of carbohydrates 2,931 cm^−1^: C−H 1,629 and 1,382 cm^−1^: C=O 1,402, 1,153, and 1,078 cm^−1^: C−O 1,238, 1,201, 1,105, and 1,031 cm^−1^: O−H	Sun W. et al. (2025)
	AP	6 h	100 °C	1:20 w/v	7.85%	12.733, 127.980, and 10.051 kDa	Ara: Rha: Gal: Glu: Gal-UA = 1.8: 1: 4.5: 2.2: 4.7	N/A	Zhu et al. (2025)
	AOB1-1	9 h	80 °C	1:15 w/v	9.50%	90.800 kDa	Man, Rha, GlcA, GalA, and Glc	t-l-Ara*f*: 1,3-l-Ara*f*: 1,5-l-Ara*f*: t-d-Glc*p*: 1,4-day-Glc*p*: 1,2,4-l-Rha*p*: 1,6-day-Man*p*: t-d-Gal*p*: 1,4-day-Gal*p*: 1,3-day-Gal*p*: 1,6-day-Gal*p*: 1,4,6-day-Gal*p*: 1,3,6-day-Gal*p*: 1,3,4,6-day-Gal*p* = 12.9: 2.9: 6.7: 9.0: 1.2: 2.5: 4.0: 8.3: 5.7: 4.7: 18.7: 1.0: 20.4: 2.0	Yang et al. (2024)
	WASCP	N/A	80 °C	1:25 w/v	N/A	76.100 kDa	Fru: Glc: Gal: Ara: Rha = 1.86: 2.18: 0.98: 1.50: 1.53	Repeating unit of −4)-α-D-GalpA-(1,2)-α-L-Rhap-(1- is mostly substituted with Galp- and Araf-based sugar residues on the third and fourth position of the rhamnose sugar ring	Wang et al. (2021), Wang et al. (2020)
	AOP	N/A	N/A	1:20 w/v	N/A	152.800 kDa	Rha: Ara: Gal: Xyl: GalUA = 0.05: 1.00: 1.35: 0.54: 2.10	N/A	Zhang W. et al. (2018)
	F-AOP			1:20 w/v		181.300 kDa	Rha: Ara: Gal: GalUA: GlcUA = 0.07: 1.00: 2.18: 5.19: 0.14		
AEM (HCl)	AP	2 h	60 °C	1:15 w/v	N/A	9.500 kDa	Man: Rha: GlcA: GalA: Glc: Gal Xyl: Fuc = 0.10: 0.30: 0.09: 1.00: 0.15: 0.43: 0.51: 0.53	3,295 and 2,936 cm^-1^: O−H and C−H 1732 cm^-1^: C−O 1,613 and 1,415 cm^-1^: O−C−O and C−OH 1,048 and 1,013 cm^-1^: C−O and C−C	Zhu et al. (2024)
UCE	AOP	46 min	30 °C	1:35 w/v	3.134%	61.800 kDa	Glc: Fuc: Ara: Gal: Rha = 2.18: 1.86: 1.50: 0.98: 1.53	N/A	Zhao et al. (2012), Zhao et al. (2011)
EAE	ASDF	4 h	55 °C–60 °C	N/A	25.58%	277.000 and 6.440 kDa	Man: Rha: GalA: Glc: Gal: Ara = 19.93: 1.02: 1.94: 32.17: 1.00: 1.91	N/A	Zhang et al. (2021)

N/A: information was not available.

In addition, the extraction methods chosen by researchers for different parts of *A. officinalis* also vary. Most researchers choose to extract polysaccharides from *A. officinalis* skin powder and waste, while a few researchers choose *A. officinalis* stems. Due to the high soluble dietary fiber content in its stem, enzyme-assisted extraction (EAE) showed better adaptability in the actual extraction process (Zhang et al., 2021). First, *A. officinalis* was gelatinized at 120 °C for 15 min, after which 2.5 mL of 50,000 U/g amylase was added and digested for 1 h, and the pH of the solution was adjusted to 4.0. Afterward, 1.2 mL of 20,000 U/g cellulase was added and hydrolyzed for 2 h. Finally, 2.5 mL of 50,000 U/g neutral protease was used for 1 h of hydrolysis, and the results showed that the extraction rate reached 25.58%. In addition, the proportion of Man in the polysaccharides obtained using this method is much higher than that in other polysaccharides.

The purification process of AOPs is a multi-step, multi-method combination process, aimed at gradually removing proteins, pigments, and other small-molecule impurities from crude polysaccharides, ultimately obtaining high-purity and homogeneous polysaccharide metabolites (Chen et al., 2024; Collings et al., 2025). The researchers chose to use the classic Sevage method to remove protein. They relied on chloroform and n-butanol reagents to react with protein to generate an insoluble gel, which was then removed through centrifugation (Rodríguez-León et al., 2026). However, it should be noted that this method requires multiple repeated operations, and the reagents contain toxicity that requires attention to safety. Macroporous resin and activated carbon are the most common methods for removing pigments. Compared to activated carbon, macroporous resin, although having a weaker adsorption capacity, has better selectivity and can be reused, making it more widely used in practical operations. In addition, ion exchange chromatography and gel filtration chromatography are the main methods for fine purification and metabolite separation of polysaccharides. The two are separated based on the charge and molecular weight of the polysaccharides. Among them, ion exchange chromatography is the main method for separating acidic and neutral polysaccharides, and it is also the most commonly chosen method for researchers to purify AOPs (Prasertsri et al., 2025). As mentioned above, it is currently difficult to purify AOPs through a single purification method. Therefore, it is necessary to scientifically select and combine purification methods based on the physicochemical properties and experimental objectives of polysaccharides and construct a reasonable and effective process route.

As mentioned above, various situations may affect the chemical structure of AOPs during the extraction process. The preprocessing methods, selection of extraction sites, and differences in extraction methods impact the results most (Liang et al., 2026; Di Matteo et al., 2025). The improvement in extraction methods not only contributes to the study of the physicochemical properties and biological activity of polysaccharides but also greatly promotes the reuse of plant waste. This aligns with the concept of green chemistry and provides new ideas for the transformation of related traditional industries to a certain extent.

### 4.2 Physicochemical properties

**FIGURE 4:**
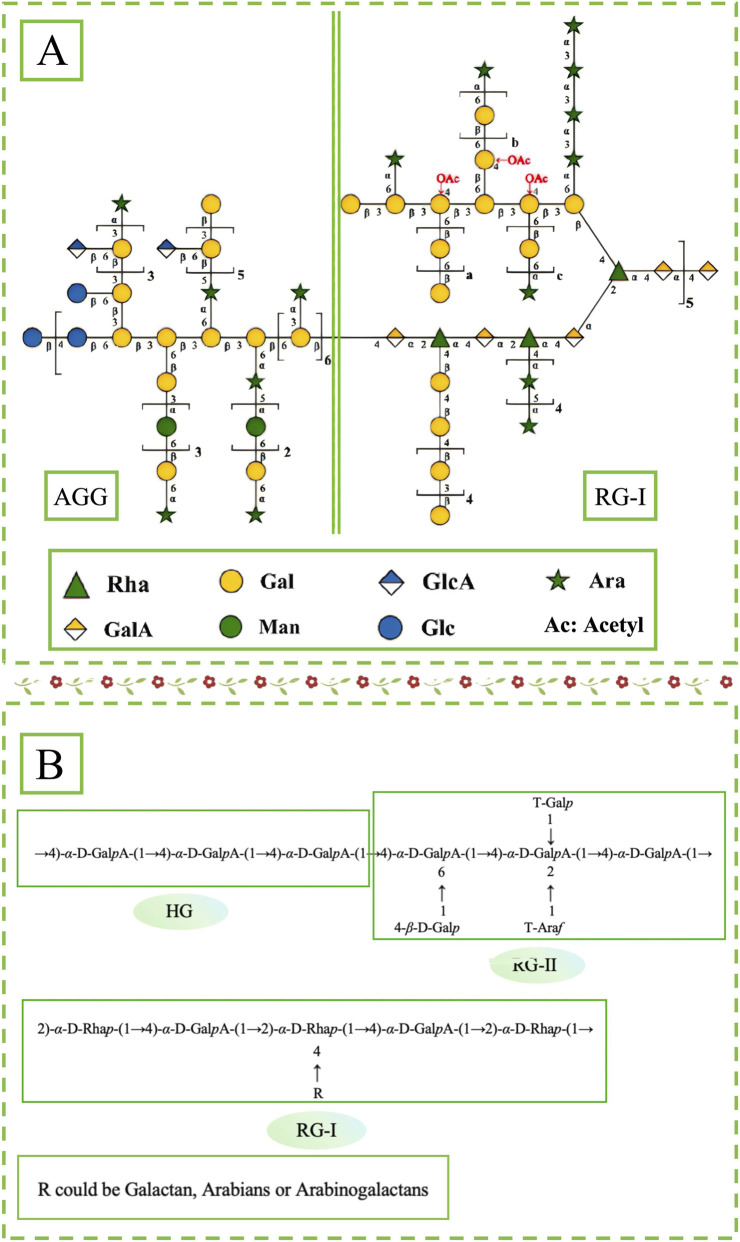
**(A)** Predicted structure of AOPB-1-1. **(B)** Proposed fragments of WASP.

The molecular weight of polysaccharides can directly affect solubility, viscosity, *in vivo* retention time, and biological activities. Its distribution can reflect the uniformity of polysaccharides and is an important indicator in the quality evaluation process. So far, the molecular weight distribution of AOPs extracted by researchers ranges from 2.612 kDa to 277 kDa. The main reason for the wide distribution of polysaccharide molecular weight is the difference in polymerization degree of different polysaccharides. As various parts of *A. officinalis* are selected as raw materials for polysaccharide extraction, the obtained polysaccharides are divided into two categories: pectin polysaccharides and cellulose polysaccharides. At the same time, the different lengths of sugar chains, branches, or cyclic structures in polysaccharides can also affect the size of their molecular weight (Wang et al., 2020). For example, its structure is mainly composed of 4-*α*-D-Gal*p*A, with *α*-L-Ara*f*, 3-*α*-L-Rha*p*, 2,4-*α*-L-Rha*p*, and 4-*β*-D-Gal*p* also being key metabolites (Figure 4). Therefore, researchers have determined that the proportion and arrangement order of different units are important factors affecting the molecular weight of AOPs.

The monosaccharide composition of AOPs extracted by researchers currently varies. This phenomenon is not uncommon and has appeared multiple times in research on other plant polysaccharides (Duan et al., 2025; Zhang et al., 2020). Researchers believe that this is due to differences in extraction methods. However, from a botanical perspective, differences in biosynthetic pathways and environmental factors are also core reasons. Cellulose is directly synthesized and arranged into the cell wall by the cellulose synthase complex in the cell membrane. Pectin is mainly synthesized in the Golgi apparatus and transported to the cell wall through vesicles. Meanwhile, the addition of each monosaccharide requires different enzymes, which explains why the monosaccharide composition of AOPs is different before and after fermentation using the same extraction steps. In addition, the cell wall metabolites of root tip cells and thick-walled tissues in *A. officinalis* undergo significant changes to adapt to growth and enhance strength. Therefore, polysaccharides from different parts of *A. officinalis* will significantly differ in various aspects.

The type, connection order, and branching degree of glycosidic bonds together determine polysaccharides’ advanced three-dimensional structure and physicochemical properties. It can also directly affect the drug-loading mode, targeting, and intelligent response behavior of nanoformulations. Researchers identified the glycosidic bond composition of AOPs, T-L-Ara*f*, 1, 3-L-Ara*f*, 1, 5-L-Ara*f*, T-D-Glc*p*, 1, 4-D-Glc*p*, 1, 2, 4-L-Rha*p*, 1, 6-D-Man*p*, T-D-Gal*p*, 1, 4-D-Gal*p*, 1, 3-D-Gal*p*, 1, 6-D-Gal*p*, 1, 4, 6-D-Gal*p*, 1, 3, 6-D-Gal*p*, 1, 3, 6-D-Gal*p*, 1, 3, 6-D-Gal*p*, 1, 3, 6-D-Gal*p*, and 1, 3, 6-D-Gal*p*. The ratio of 4,6-D-galactose is 12.9: 2.9: 6.7: 9.0: 1.2: 2.5: 4.0: 8.3: 5.7: 4.7: 18.7: 1.0: 20.4: 2.0 (Yang et al., 2024). The sugar composition analysis report shows that some AOPs contain two types of uronic acids (GlcA and GalA), which cannot be effectively distinguished solely through methylation analysis. The researchers determined its uronic acid content to be 11.9% using the sulfuric acid carbazole method. Before methylation analysis, researchers conducted uronic acid reduction, consistent with monosaccharide composition analysis, with a uronic acid reduction rate of 99.0%. Compared with that before reduction, the content of T-D-Glc*p* and 1,4-D-Gal*p* in the reduced *A. officinalis* polysaccharides significantly increased from 1.3% and 2.6% to 9.0% and 5.7%, respectively, indicating the presence of uronic acid in these bonds. The ratio of 1,4-D-Gala*p* to 1,2,4-L-Rha*p* is approximately 1, confirming that they originate from the rhamnogalactose-I (RG-I) domain of AOPs (Yang et al., 2024). Meanwhile, researchers found that most of the sugar residues based on rhamnose originate from the RG-I structure in another type of pectin polysaccharide. In the main chain of RG-I, the repeating units of -4)-*α*-D-Gal*p*A-(1,2)-*α*-L-Rha*p*-(1) are mainly replaced with Gal*p* and Ara*f* glycosides at the third and fourth positions of the rhamnose ring, respectively, which is consistent with the results obtained by researchers through nuclear magnetic resonance analysis (Wang et al., 2020).

There are various reasons for the structural differences in *A. officinalis* polysaccharides, and the structural differences between different types of polysaccharides are significant. Different extraction methods and different parts of polysaccharide sources directly determine whether the extracted AOPs are pectin polysaccharides or cellulose polysaccharides. This is also the main reason why the monosaccharide composition of AOPs currently obtained varies. Whether there are significant differences in the chemical structures of AOPs from different varieties of *A. officinalis* is also a matter of concern. In future research, the focus should be on the influence of *A. officinalis* varieties on the chemical structure of AOPs while verifying existing conclusions to obtain more valuable information.

## 5 Health benefits

### 5.1 Regulating gut microbiota

**FIGURE 5:**
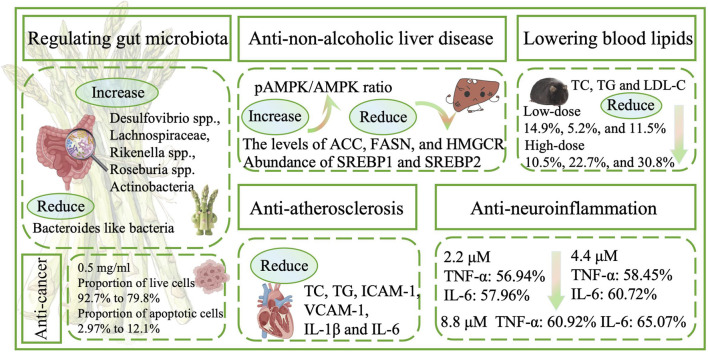
The health benefits of *Asparagus officinalis* L. polysaccharides.

In traditional medicine, *A. officinalis* is considered to have good anti-inflammatory effects, and its rich dietary fiber makes it particularly effective in regulating gut microbiota. For this reason, researchers often pay great attention to diseases that can be treated by regulating the direction of gut microbiota during the experimental process (Brown et al., 2025; Zhang Q. et al., 2025). The regulation of gut microbiota is considered the most important health benefit of AOPs for the human body. Researchers found that the effects of AOPs in treating non-alcoholic liver disease, anti-atherosclerosis, and lowering blood lipids depend on their role in regulating intestinal flora (Table 3; Figure 5).

**TABLE 3:** Health benefits of *Asparagus officinalis* L. polysaccharides.

Health benefits	Polysaccharide name	*In vitro* or *in vivo*	Indicated concentrations	Models/test system	Action or mechanism	Ref
Anti-non-alcoholic liver disease	AP	*In vivo*	0.5% and 3% AP	C57BL/6J mice	1. Increased the abundance of *Romboutsia* and *Lactobacillus* while decreasing the abundance of *Escherichia Shigella* 2. Restored pAMPK/AMPK levels. Reduced the abundance of SREBP1 and SREBP2. Reduced the expression of FASN, ACC, and HMGCR in the liver	Sun L. et al. (2025)
Anti-atherosclerosis	AP	*In vivo*	150 mg/kg	C57BL/6J mice	1. Weaken *aortic* plaques. Reduce TC, TG, ICAM-1, VCAM-1, IL-1β, IL-6, and TNF -*α* 2. Slightly *increased* the abundance of *Bacteroidetes* and significantly reduced the abundance of *Firmicutes* 3. The *abundance* of *s_Bifidobacheter_pseudolangum* and *C. stationis* significantly increased	Zhu et al. (2025)
Lowering blood lipids	ASDF	*In vivo*	400 and 800 mg/kg	Kunming mice	1. Liver index decreased by 4.31% and 4.96%, respectively 2. At 400 mg/kg, the levels of TC, TG, and LDL-C decreased by 14.9%, 5.2%, *and* 11.5%, respectively. At 800 mg/kg, the levels of TC, TG, and LDL-C decreased by 10.5%, 22.7%, and 30.8%, respectively	Zhang et al. (2021)
Anti-neuroinflammation	AOBP-1–1	*In vitro*	2.2, 4.4, and 8.8 μM	BV2 microglia cells	1. Reduced TNF-α *levels* by 56.94%, 58.45%, and 60.92% and reduced IL-6 levels by 57.96%, 60.72%, and 65.07% 2. Inhibit *the* expression of inflammatory genes such as COX2, iNOS, TNF-α, IL-1β, and IL-6	Yang et al. (2024)
Cytotoxic and pro-apoptotic effects	AOP, DP-AOP, AOP-4, AOP-6, and AOP-8	*In vitro*	10 mg/ml	HeLa cells and BEL-7404 cells	1. At 10 mg/mL, the inhibition rates of HeLa cells were 54.64%, 67.86%, 83.96%, 81.03%, and 72.05% 2. At 10 mg/mL, the inhibition rates on BEL-7404 cells were 57.31%, 57.73%, 55.39%, 62.28%, and 54.41%	Zhao et al. (2012)
	AP	*In vivo*	0.25 and 0.5 mg/ml	C57BL/6 mice	1. Reduced *MSC*-2 cell viability and increased lymphocyte viability in a dose-dependent manner 2. Promote MDSC cell apoptosis in a dose-dependent manner 3. The level of phospho-NF-κB p65 increased	Zhang Z. et al. (2018)
​	ASP	*In vitro*	2.5, 5, and 10 mg/ml	Hepatocellular carcinoma cells	1. SK-Hep1 cells: IC_50_ = 4.76 mg/ml, Hep-3B Cells: IC_50_ = 8.18 mg/ml 2. Inhibition of *upregulation* of HIF-1*α* and VEGF in HCC cells 3. Inhibition *of* protein expression of p-AKT, p-mTOR, and p-ERK	Chen et al. (2024)
Immune regulation	WASP	*In vitro*	50, 100, and 200 μg/ml	RAW264.7 cells	Promote the secretion of RAW264.7 cells, as well as the mRNA expression levels of TNF-α, IL-10, and IL-6, and enhance immune response	Wang et al. (2020)
	WASP-40, WASP-50, WASP-60, WASP-70, and WASP80	*In vitro*	20, 50, 100, 200, 400, and 800 μg/ml	RAW264.7 cells	1. WASP-40 can promote the production of IL-6 2. Except for WASP-80, all can promote the production of TNF-α 3. At a *concentration* of 200 μg/mL, IL-10 cytokine levels can be increased	Wang et al. (2021)

#### 5.1.1 Anti-non-alcoholic liver disease

**FIGURE 6:**
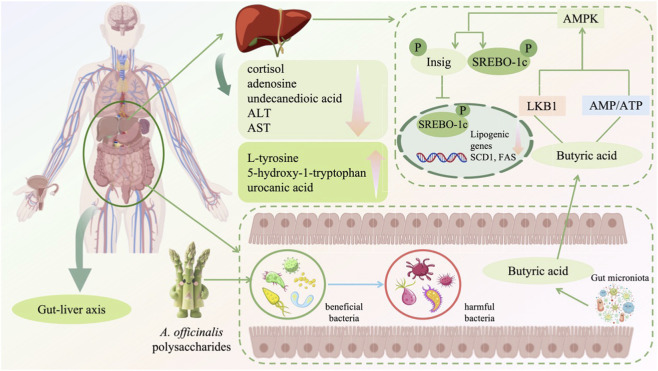
Potential mechanism of action of *Asparagus officinalis* L. polysaccharides in anti-non-alcoholic liver disease.

The researchers used male C57BL/6J mice for *in vivo* experiments, in which non-alcoholic liver disease was induced by a high-fat diet, leading to liver lipid accumulation and inflammatory responses. The mice were randomly divided into five groups: normal group, high-fat-diet group, 0.5% and 3% AOPs intervention group, and 3% AOPs and 0.5% polyene phosphatidylcholine intervention group. The fecal samples of mice were collected in the last 2 weeks of the experiment. After the experiment, the liver and colon of mice were removed immediately for cryopreservation for subsequent analysis. After treatment with AOPs, the levels of blood lipids and liver lipids in mice were significantly reduced, and the liver injury and inflammation in mice were also significantly improved. In subsequent studies, researchers have demonstrated that the intervention of AOPs can regulate the AMPK/SREBP signaling pathway, increase the pAMPK/AMPK ratio, and reduce the abundance of SREBP1 and SREBP2 in the nucleus, thereby further reducing the levels of acetyl coenzyme A carboxylase (ACC), fatty acid synthase (FASN), and 3-hydroxy-3-methylglutaryl-coenzyme A reductase (HMGCR) and affecting liver lipid synthesis (Sun W. et al., 2025). In addition, AOPs can enhance the integrity of the intestinal barrier, reduce plasma endotoxin levels, and inhibit the endotoxin/TLR4/NF-κB signaling pathway. This led to a decrease in the levels of inflammatory factors in the plasma, achieving the effect of alleviating inflammation in mice. It is worth noting that researchers concluded based on the results of the fecal microbiota transplant (FMT) experiment that the changes in gut microbiota induced by AOPs also improved lipid metabolism through AMPK/SREBPS and LPS/TLR4/NF-κB pathways (Figure 6). Due to the diversity of the mouse gut microbiota, significant improvement was observed after intervention treatment with AOPs. Therefore, researchers have concluded that AOPs exert a therapeutic effect on non-alcoholic liver disease by altering the composition of the gut microbiota rather than increasing species richness.

Based on the above results, researchers conducted a detailed analysis of gut microbiota composition in mice. The intervention of AOPs significantly increased the abundance of *Desulfovibrio* spp., *Lachnospiraceae*, and *Rikenella* spp. while reducing the abundance of *Faecalibaculum* spp. and *Proteus* spp. In addition, the increased abundance of butyric acid-producing bacteria *Lachnospiraceae*, *Rikenella* spp., and *Roseburia* spp (Sun L. et al., 2025) indicates that AOPs have a probiotic function of promoting butyric acid-producing bacteria proliferation (Zhang et al., 2026).

#### 5.1.2 Anti-atherosclerosis activity

**FIGURE 7:**
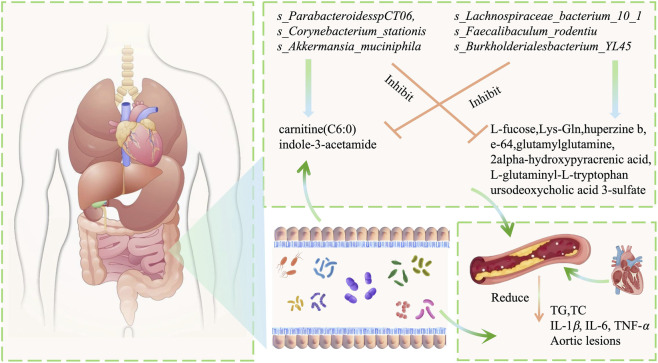
Potential mechanism of the anti-atherosclerosis effects of *Asparagus officinalis* L. polysaccharides.

When the researchers studied the mice induced with a high-fat diet, male ApoE^−^/^−^C57BL/6J mice were selected for *in vivo* experiments; mice fed a standard laboratory rodent feed served as the control group, and mice fed a high-fat diet served as the model group. Simultaneously, AOPs were added to the control diet and the high-fat diet at a dose of 150 mg/kg/day. The experiment lasted for 12 weeks. After that, the aortic tissue was dissected from the aortic arch to the thoracic aorta to ensure that all samples were collected in the same anatomical area. At the same time, cecal and colorectal contents were collected to analyze the intestinal microbiota of the mice. The researchers found that the mice exhibited more severe aortic plaques than the control mice, and the administration of AOPs reduced the effects of these plaques. The researchers detected the expression levels of genes such as total cholesterol (TC), triglyceride (TG), ICAM-1, VCAM-1, IL-1β, and IL-6, all of which were significantly reduced. Simultaneously, researchers also measured serum AST, ALT, and CREA levels to evaluate the safety of AOPs in important organs. Among them, AST, ALT, and CREA levels in mice induced by a high-fat diet were significantly increased, while AOPs significantly decreased them (Zhu et al., 2025). Therefore, researchers believe that AOPs can play a role in improving atherosclerosis and inflammation of important organs (Figure 7). In addition, researchers studied the effect of AOPs on the gut microbiota composition of mice through 16S rRNA sequencing and detected microbial diversity. Among them, the diversity of gut microbiota in mice induced by a high-fat diet significantly decreased, while the diversity increased after treatment with AOPs. In the subsequent analysis of the gut microbiota of mice at the phylum and genus levels, the abundance of *Bacteroides* and *Actinobacteria* in the gut of mice induced by a high-fat diet decreased. In contrast, the abundance of bacteria and Proteobacteria at the phylum and genus levels increased. After treatment with AOPs, the abundance of actinomycetes increased, with *s_Corynebacterium-stationis (Actinobacteria)* being the dominant species. Researchers have demonstrated through data analysis that an increase in the ratio of gut microbiota to Bacteroidetes is positively correlated with cardiovascular disease. In addition, researchers believe that tryptophan metabolism derived from intestinal microflora is directly related to atherosclerosis. It is speculated that the protective effect of AOPs on atherosclerosis may be closely related to tryptophan metabolism of intestinal microflora.

#### 5.1.3 Lowering blood lipids

According to the existing literature, AOPs can effectively inhibit the weight gain of mice induced by a high-fat diet. The researchers randomly divided Kunming mice into four groups for *in vivo* lipid-lowering experiments. The experiment lasted for 7 weeks. The control group was given a normal diet, and except for the high-fat diet group, the other two groups of mice were administered 400 mg/kg and 800 mg/kg AOPs and the same high-fat diet every day, as the low-dose group and the high-dose group, respectively. After the experiment, the liver was collected for weighing, and fecal samples were also collected. In addition, the liver indices of mice in the high-dose group administered with 800 mg/kg AOPs per day and the low-dose group administered with 400 mg/kg AOPs per day decreased by 4.96% and 4.31%, respectively, indicating that AOPs can effectively alleviate the adverse effects of hyperlipidemia on the liver. At the same time, researchers found that a high-fat diet significantly increased the levels of TC, TG, low-density lipoprotein cholesterol (LDL-C), and high-density lipoprotein cholesterol (HDL-C) in mouse serum, indicating interference with lipid metabolism in mice. After low-dose AOP treatment, TC, TG, and LDL-C levels in mice decreased by 14.9%, 5.2%, and 11.5%, respectively. After high-dose AOP treatment, the levels of TC, TG, and LDL-C in mice decreased by 10.5%, 22.7%, and 30.8%, respectively (Zhang et al., 2021). Researchers have concluded that AOPs can exert a dose-dependent lipid-lowering effect, also indicating that AOPs have great potential in functional food development and potential drug screening.

In subsequent studies on gut microbiota, researchers found that after feeding mice with a high-fat diet, the relative abundance of filamentous bacteria significantly decreased from 78.9% to 45.7%. The gene frequency of Firmicuts, the second most abundant system type, increased from 14.9% to 32.0%. Treatment with low-dose AOPs and high-dose AOPs reduced the increased abundance of Firmicuts to 28.1% and 20.9%, respectively. In addition, AOPs can also reduce the relative abundance of *Proteus* and *Staphylococcus* in a dose-dependent manner. At the same time, researchers concluded through data analysis that *Staphylococcus aureus* is significantly positively correlated with TC and LDL-C levels.

### 5.2 Anti-neuroinflammation

Neuroprotection plays an irreplaceable role in maintaining brain health, preventing diseases, and ensuring the quality of human life. In recent studies, AOPs have been shown to exert neuroprotective effects through anti-neuroinflammation. In other words, AOPs can have both preventive and reparative neuroprotective effects. This is also in line with the core of neuroprotection, which strives to maintain the integrity of the number and function of neurons as much as possible.

Researchers chose *in vitro* experiments to study the anti-neuroinflammatory activity of AOPs. During this period, the researchers evaluated the effect of AOPs on the viability of BV2 cells using the MTT method and the effect of AOPs on the release of NO in LPS-stimulated BV2 cells using Griess reagent. Studies have shown that AOPs can significantly reduce the content of inflammatory cytokines in endotoxin-stimulated BV2 cells, including COX2, iNOS, TNF-α, IL-6, and IL-1β. The experimental results showed that AOPs could significantly inhibit endotoxin-induced NO production at 1.1, 2.2, 4.4, and 8.8 μM. In addition, at 2.2 μM, the reduction in NO is equivalent to the therapeutic effect of 5 μM MINO. In subsequent studies, AOPs were found to reduce TNF-α levels by 56.94%, 58.45%, and 60.92%, along with IL-6 levels by 57.96%, 60.72%, and 65.07% at 2.2, 4.4, and 8.8 μM. At 2.2 and 8.8 μM, AOPs showed better therapeutic effects than 5 μM MINO (Yang et al., 2024). This indicates that AOPs can exert anti-neuroinflammatory effects by reducing the release of inflammatory cytokines. In addition, adding 8.8 μM AOPs resulted in a 38.0% decrease in IL-1β levels and a 45.4% decrease in COX2 levels, both of which were superior to the therapeutic effect of MINO. This experiment demonstrates that AOPs have great potential in anti-neuroinflammation, which is consistent with the traditional medicinal use of *A. officinalis*. Therefore, AOPs have significant advantages in the development of anti-neuroinflammatory drugs. In subsequent research, particular attention should be paid to the stability of AOPs during treatment, with the aim of achieving clinical translation as soon as possible.

### 5.3 Cytotoxic and pro-apoptotic effects

In early experiments, researchers first used HeLa cells and BEL-7404 cells to evaluate the inhibitory effect of AOPs *in vitro*. Experiments are more inclined to evaluate the safety of drugs. Due to the absence of a biological environment, it is impossible to predict the absorption, distribution, and metabolism of AOPs *in vivo*. Therefore, although many valuable data have been provided for the follow-up study, the degree of research is relatively shallow, and the mechanism of action has not been investigated. After that, some researchers used C57BL/6 mice to establish a colon tumor model and confirmed that AOPs can play an anti-colon cancer effect by inhibiting myeloid-derived suppressor cells (MDSCs). Researchers isolated MDSCs from the spleen of mice with colon cancer to establish an *in vitro* model to evaluate the therapeutic effect of AOPs. During the experiment, researchers found that AOPs reduced the number of MDSCs (Gr1^+^, CD11b^+^) in a dose-dependent manner and achieved optimal results at a concentration of 0.5 mg/ml. Its IC_50_ value is 0.4919 mg/ml. In addition, while detecting the cytotoxicity of AOPs, researchers found that AOPs can reduce the cellular activity of MSC-2 and increase the activity of lymphocytes. Based on this, researchers conducted typical quadrant analysis of adherent spleen cells in mice with MC38 tumor using AOPs at concentrations of 0, 0.25, and 0.5 mg/ml in subsequent studies. The proportion of live cells in the 0.5 mg/ml AOPs treatment group decreased from 92.7% to 79.8%, and the proportion of early apoptotic cells increased from 2.97% to 12.1%, showing a dose-dependent effect (Zhang W. et al., 2018; Zhao et al., 2012). In summary, AOPs can significantly inhibit the proliferation of MDSCs and induce cell apoptosis in a toll-like receptor 4-dependent manner. Subsequent studies have shown that AOPs can upregulate the expression of Bax and Caspase-9 and inhibit the expression of Bcl2, indicating that AOPs can induce apoptosis of MDSCs through endogenous pathways. However, this method does not belong to the traditional anti-cancer experiment *in vivo*, and there is no clear grouping and specific dose arrangement of AOPs. Therefore, this experiment is more inclined to study the cytotoxicity and pro-apoptotic effect of AOPs.

At the same time, AOPs have also shown great potential for development in anti-liver cancer research. Researchers used CCK-8 to detect the inhibitory effect of AOPs on liver cancer cells at concentrations of 10, 5, and 2.5 mg/ml. The results showed that AOPs could effectively inhibit the proliferation, migration, invasion, and angiogenesis of SK-Hep-1 and Hep-3B cells induced by hypoxia and showed a dose-dependent relationship. Their IC_50_ values are 4.76 mg/ml and 8.18 mg/ml, respectively. Meanwhile, researchers believe that the inhibitory effect of AOPs may be related to their downregulation of HIF1α and VEGF expression in SK-Hep-1 and Hep-3B cells under hypoxic conditions. In addition, signal pathway studies have shown that AOPs significantly downregulate the expression of hypoxia-induced p-AKT, p-mTOR, and p-ERK while having little effect on AKT, mTOR, and ERK expression. In the subsequent SK-Hep-1 nude mouse transplant tumor model, researchers further confirmed that AOPs may exert inhibitory effects on transplant tumors by blocking the MAPK and PI3K signaling pathways, downregulating the expression of HIF1*α* and vascular endothelial growth factor.

### 5.4 Immune regulation

To explore the effect of AOPs on immune activity, researchers used different concentrations of AOPs and observed their effect on the proliferation of RAW264.7 macrophages within 24 h. The experiment was divided into nine groups, namely, a blank group, a 100 μg/ml negative control group of Arabic gelatin, a 2 μg/ml LPS positive control group, and 20, 50, 100, 200, 400, and 800 μg/ml AOP experimental groups. CCK-8 and neutral red staining assays were used to evaluate the effects of different components on RAW264.7 macrophages. At a concentration of 200 μg/mL, the phagocytic ability of macrophages was significantly enhanced, and AOPs did not exhibit cytotoxicity within this concentration range. In subsequent studies, researchers demonstrated that AOPs regulate the immune response of RAW264.7 macrophages by increasing the release of immune factors (IL-6, TNF-α, and IL-10) and promoting their gene expression. In addition, researchers also investigated the effect of fermentation steps on the immunomodulatory activity of AOPs. Researchers conducted studies using cyclophosphamide-induced mouse immunosuppression models. At a dose of 200 mg/kg, the F-AOPs significantly increased the levels of IgA and IgG, while at a dose of 100 mg/kg, the level of IFN-*γ* was significantly increased, and the level of IL-10 was decreased (Wang et al., 2021; Wang et al., 2020). This indicates that F-AOPs can have a consistent stimulating effect on the humoral immunity suppressed by cyclophosphamide, and compared with AOPs, F-AOPs have a better effect on enhancing the immune function of Th1 cells. As mentioned above, the fermentation process can promote the role of polysaccharides in immune regulation. However, the specific promotion ratio and effective concentration range still need to be focused on in subsequent research.

## 6 Structure–activity relationships

Most AOPs are miscellaneous polysaccharides, mainly divided into pectin polysaccharides and cellulose polysaccharides, among which there are also some polysaccharide complexes (Mongraykang et al., 2025). For example, the AOPs extracted by Yang et al. (2024) are a pectin arabinose galacturonate complex. Its structure contains a large amount of uronic acid, which has a significant negative charge at physiological pH (Yang et al., 2024). Previous studies have shown that biological activity of polysaccharides is related to their charge density. Therefore, researchers believe that the enhanced binding affinity between AOPs and related proteins results in good anti-neuroinflammatory activity, mainly due to their acidic groups.

In terms of monosaccharide composition, AOPs contain various monosaccharides and monosaccharide derivatives such as Rha, Ara, GlcN, Gal, Glc, GlcNAc, Gal-UA, Xyl, Man, Fru, and Rib. The wide variety of monosaccharides creates complex structure–activity relationships. In addition, the abundant monosaccharides have constructed more complex and highly branched polysaccharide structures. This also suggests that AOPs have a larger specific surface area and terminal residues, which are key sites for the interaction between AOPs and biomolecules. In addition, the diversity of monosaccharides results in a more advanced three-dimensional structure, which is also crucial for AOPs to be recognized by and function within the immune system.

Meanwhile, the wide variety of monosaccharides directly promotes the role of gut microbiota. Different gut microbiota have different carbohydrate-active enzymes, which can only perform specific degradation on specific sugar chain structures. More diverse and complex sugar chain structures can also interact with various bacterial communities (Chang et al., 2025; Sun W. et al., 2025). Complex heteropolysaccharides can meet the growth needs of a wider range of bacterial communities (Oyovwi et al., 2025). Meanwhile, different bacterial strains collaborate to degrade the same polysaccharide molecule, forming a mutually beneficial relationship that contributes to the stability of the gut microbiota ecosystem.

It is worth noting that the physicochemical properties, such as monosaccharide compositions and molecular weight of F-AOPs, undergo significant changes, and researchers have confirmed that the immune activity of F-AOPs is stronger than that of unprocessed AOPs (Zhang Z. et al., 2018; Zhang W. et al., 2018). In addition, researchers have found through serum metabolomics studies that F-AOPs can effectively alleviate liver damage caused by cyclophosphamide. Among them, the inhibitory effect of F-AOPs on liver biochemical markers and pro-inflammatory cytokines is more significant than that before processing. Meanwhile, F-AOPs also showed better effects in promoting GSH biosynthesis, normalizing the expression levels of bile acid receptors and key enzymes involved in bile acid synthesis. In addition, F-AOPs exhibit a more advantageous effect in increasing SCFA production than unprocessed AOPs (Zhang et al., 2020). Therefore, researchers believe that this is the key factor leading to stronger hepatoprotective activity of F-AOPs.

Properties such as molecular weight distribution, solubility, viscosity, three-dimensional conformation, and biological stability, all depend on molecular weight (Ganie et al., 2026). At the same time, the molecular weight of polysaccharides also affects receptor binding affinity, cell uptake efficiency, and metabolic pathways *in vivo*. The molecular weight distribution of AOPs ranged from 2.612 to 277 kDa. Generally, polysaccharides with a molecular weight of less than 10 kDa are considered more easily absorbed or penetrated by cells and may show stronger antioxidant or anti-inflammatory abilities. Polysaccharides with molecular weight in the range of 10–500 kDa have advantages in immune regulation and anti-tumor activity. According to the latest research report, moderate reduction in molecular weight can optimize the spatial conformation of polysaccharide, increase the accessibility of active sites, fine-tune, and optimize its immune regulation function. However, at present, the molecular weight of AOPs is relatively low, which is mainly based on previous observations. Therefore, it is necessary to pay more attention to pharmacological experiments in the follow-up study for verification. In addition, most of the glycosidic bonds in AOPs are *α*-glycosidic bonds, and the connection length is usually short, which can promote the tight binding between sugar molecules and improve the stability of the whole molecule. At the same time, *α*-glycosidic bonds can be hydrolyzed by special enzymes such as acid and *α*-amylase, and the hydrolysis rate is slower than that of *β*-glycosidic bonds. However, it can provide longer stability in actual product development (Zhong et al., 2025). It is worth noting that AOPs present dispersed spherical particles and an ordered triple helix conformation in three-dimensional conformation. PRRS on the surface of macrophages can effectively recognize this structure, leading to the significant upregulation of NLRP3 inflammasome expression and typical pyrolysis mediated by NLRP3 inflammasome (Xu et al., 2026). This shows that AOPs have great research and development space in anti-inflammatory and immune regulation. Simultaneously, this also provides strong support for the investment of follow-up researchers in the targeted screening and optimization of functional polysaccharides (Shen et al., 2026; Zhang Z. et al., 2025). In general, the research status of structure–activity relationships of AOPs mainly focuses on monosaccharide composition. Although the research conclusions on molecular weight distribution, glycosidic bond structure, and three-dimensional structure are one-sided, they still have great development potential.

## 7 Preparation and applications of nanocrystals

### 7.1 Preparation

Nanomaterials have undoubtedly become an important research topic in recent years, receiving significant attention in the fields of materials, the food industry, and medicine. Some researchers used *A. officinalis* stems as raw materials, dispersed them in water at a ratio of 1:25, adjusted the pH to 12, and heated the mixture to 90 °C for 165 min. After that, the sample was filtered and re-dispersed in water at a ratio of 1:2.32. The pH was adjusted to 7, and the sample was filtered after bleaching to obtain cellulose-based fibers. Afterward, it was dispersed in 60 wt% sulfuric acid at 1:25, stirred, and hydrolyzed for 1.5, 2, 2.5, 3.0, and 3.5 h to obtain five different sizes of cellulose nanocrystal samples. As the hydrolysis time increased, researchers observed that the shape of cellulose particles became closer to needle-like and exhibited obvious needle-like features after 2.5 h, which is believed to be the result of removing amorphous cellulose. The size of its cellulose nanocrystals ranges from 178.2 to 261.8 nm, with a crystallinity of 72.4%–77.2%. Meanwhile, during the period of 1.5–2.5 h, the crystallinity increased from 72.35% to 77.18%, while after exceeding 2.5 h, the crystallinity decreased (Villanueva-Suárez et al., 1999). In addition, nanotube water dispersion also exhibits typical shear thinning behavior. In a 30:70 palm oil: water model solution, researchers observed stable Pickering emulsions ranging from 1 to 10 μm using optical and confocal laser scanning microscopy. In addition, the cellulose nanocrystals prepared under a hydrolysis time of 3 h have relatively effective emulsifying ability for palm oil droplets.

### 7.2 Applications

Cellulose nanocrystals have been applied to varying degrees in different fields. Commonly, composite coatings made from it can be applied to preserve berries (Canazza et al., 2025; Özden et al., 2025; Begum et al., 2026). They not only address the problems of perishability and dehydration associated with traditional packaging but also help prevent mold contamination (Tang et al., 2026). At the same time, it can also address the environmental pollution problems caused by traditional, difficult-to-degrade materials. In addition, probiotic microcapsules made of cellulose nanocrystals are also a major development hotspot in drug development (Wen et al., 2025). The composite film made of nanocrystals forms a dense network through hydrogen bonding. It can effectively isolate stomach acid and protect medication (Gorina et al., 2025). Moreover, it can begin to degrade in the small intestine and colon, allowing for precise release of probiotics and forming targeted effects (Fakhlaee et al., 2026; Luo et al., 2026). In addition, developing and applying wound dressing hydrogels have also brought great convenience (Solhi et al., 2025; Ying et al., 2025). Chronic wounds are difficult to heal and susceptible to infection. The hydrogel added with cellulose nanocrystals can form a double network with cross-linking agents, which can load antibacterial agents into the hydrogels (Gärber et al., 2025). Compared with ordinary hydrogels, the most notable feature is that the addition of cellulose nanocrystals significantly improves the mechanical strength of hydrogels, making them difficult to destroy (Kaewprachu et al., 2026; Zhu et al., 2026). In summary, adding cellulose nanocomposites transforms the originally inert material into an active participant in biological processes (Zhao et al., 2025). At the same time, it is possible to fully utilize the by-products of *A. officinalis* to create greater economic value. This also provides a clear roadmap for future research.

## 8 Nutritional value analysis and food industry applications

### 8.1 Nutritional value analysis

As the main edible part of *A. officinalis* is its tender stem, almost all nutrition and food industry research currently revolves around this. *A. officinalis* has been widely developed and applied due to its rich nutritional value and health benefits (Ibrahim et al., 2025; Li T. et al., 2025). The nutritional value of *A. officinalis* mainly manifests as low-calorie, high-fiber, and contains various bioactive substances (Li Y. et al., 2025; Xie et al., 2025). In terms of macronutrients, *A. officinalis* has a water content of more than 92% and a calorie content of approximately 20 kcal/100g, making it an undisputed low-calorie food (Liu et al., 2024). The proportion of protein can reach 2.2%, which is relatively high in vegetables. Meanwhile, *A. officinalis* also contains approximately 2% dietary fiber, mostly water-soluble, which can effectively promote intestinal health and maintain blood sugar stability (Wang et al., 2016). In terms of trace elements, *A. officinalis* is rich in vitamins, especially vitamin K and vitamin B9. Therefore, *A. officinalis* is also very suitable for consumption during pregnancy (Jana et al., 2025; Kabir et al., 2024). In addition, *A. officinalis* contains various minerals, including potassium, phosphorus, calcium, magnesium, and selenium, which can effectively maintain fluid balance and blood pressure stability and have great benefits for bone health. It is worth noting that the three common varieties of *A. officinalis*—green, white, and purple—differ in nutritional composition. Green *A. officinalis* typically contains higher levels of antioxidants such as flavonoids than other varieties, while white *A. officinalis* has greater mineral and vitamin E content. Purple *A. officinalis* is rich in anthocyanins, with high sugar content and low cellulose, making it more suitable for raw consumption. Additionally, *A. officinalis* contains relatively high levels of purines compared to other vegetables. Patients with high uric acid or gout should limit their daily intake to no more than five spears. In short, *A. officinalis* is a kind of super food that is suitable for the elderly and children, as well as patients with diabetes or hypertension and pregnant women. Therefore, to better process and apply *A. officinalis* and create more economic value, the modern *A. officinalis* industry is presenting a development trend that is guided by nutrition and health, supported by high-tech technology, and achieves high value and zero waste throughout the entire industry chain.

### 8.2 Food industry applications

*A. officinalis* is rich in nutritional value but difficult to preserve, which has caused certain difficulties in its commercialization and industrial processing. Therefore, research on the preservation technology of *A. officinalis* is the primary issue that needs to be addressed in industrial processing (Tengsuthiwat et al., 2024; Ying et al., 2024). At present, researchers have adopted two technologies, physical preservation and chemical preservation, to address the issues of water loss and fibrosis, decreased nutritional quality, and microbial decay of *A. officinalis* after harvesting (Zhang et al., 2024). In terms of physical preservation, a low-temperature cold chain is the key, and rapid precooling can effectively alleviate the dehydration and fibrosis of *A. officinalis*. At the same time, adjusting the concentration of oxygen and carbon dioxide in the packaging bag can inhibit the growth of microorganisms in *A. officinalis*, which is currently the main technology (Chileh-Chelh et al., 2024; Yadav et al., 2024). In addition, in the production of high-value-added products, depressurization storage is also a commonly used technology by producers. By reducing air pressure to promote the discharge of ethylene and metabolic waste, although the equipment cost is high, it can greatly extend the storage time of *A. officinalis*. In terms of chemical preservation, edible films such as chitosan and sodium alginate are used to coat the surface of *A. officinalis*, which is currently a common preservation technique (Dos Santos-Silva et al., 2024; Olas, 2024).

**FIGURE 8:**
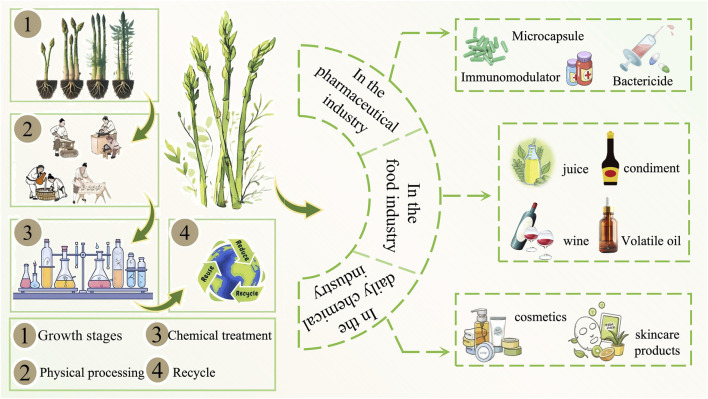
Aspar*agus officinalis* L. polysaccharide-related products.

The current mainstream research focuses on using natural plant extracts such as tea polyphenols and essential oils for preservation, which is also in line with the trend of green and safe development in the contemporary food industry. The traditional *A. officinalis* products are mainly canned *A. officinalis* and freeze-dried *A. officinalis* powder. Although there is a significant loss in taste and flavor, it has received high praise in the market as a seasoning. Currently, the mainstream direction is how to create high-value-added products while improving industrial efficiency and reducing waste (Li et al., 2023; Wang F. et al., 2025). *A. officinalis* juice is a representative product in this direction, and the key is to preserve the flavor to the greatest extent possible while preventing juice browning and sedimentation through processes such as juicing, enzymatic hydrolysis, and filtration. At the same time, the key selling point is to maximize the retention of functional metabolites such as polysaccharides, saponins, and dietary fiber in *A. officinalis* in the product, while continuing the health benefits of *A. officinalis* itself. In addition, the metabolite beverage developed by compounding with other fruits, vegetables, grains, and even botanical drugs also conforms to the concept of healthy eating for contemporary consumers (Figure 8). Research on the food industry of *A. officinalis* is a multidisciplinary field, covering multiple disciplines such as food science, nutrition, and biochemistry. Deeply exploring the health value of *A. officinalis* and promoting the transformation and upgrading of the *A. officinalis* industry from primary agricultural products to a high-tech healthy food industry has become a current research focus.

## 9 Discussion and prospect

At present, the research on AOPs focuses on three aspects: extraction and purification methods, structural characterization, and biological activity evaluation, and it has a preliminary understanding of the effect of extraction methods on the structure and biological activity of AOPs, the mechanism of biological activities, and potential structure–activity relationships. Combined with the previous review of *Asparagus* genus polysaccharides, the changes in chemical structures and biological activities of AOPs before and after fermentation and the application of its processed products in the biomedicine and food industries are the main differences between AOPs and other plant polysaccharides. It is noteworthy that, in terms of the current research report, the regulation of AOPs on intestinal flora is the focus of researchers. Still, there is no similar trend in the study of polysaccharides from other plants of the *Asparagus* genus.

In terms of extraction methods, current research shows an obvious trend of technical iteration, that is, from a single method to a combination of multiple methods, from empirical optimization to response surface and other statistical modeling optimization. However, the effect of different extraction methods on the chemical structures of AOPs lacks a systematic comparative study. At the same time, whether there are significant differences in polysaccharides among different *A. officinalis* varieties, these issues are worthy of further discussion by researchers, which is also a unique research direction of *A. officinalis*, different from other plants of the *Asparagus* genus. In terms of chemical structures, most studies still remain at the level of primary structure description, and the characterization of high-level structure is insufficient. The conclusion of structure–activity relationships mostly depends on the research experience of other plant polysaccharides. At present, the research trend of polysaccharides’ bioactivity is gradually turning to the in-depth study of biological functions, such as receptors and signal transduction, rather than simple exploration. Therefore, the chemical structure information of polysaccharides will be more important than before. To address the above limitations, researchers must adopt a multi-technology, multi-dimensional, and interdisciplinary integration strategy. Advanced instruments and technologies need to be utilized for precise preparation and separation, along with chemical and computational biology methods for molecular simulation and modeling. Technological limitations can be effectively overcome only through systematic methods and multiple fusion strategies.

In addition, the residue extracted from AOPs can also generate significant environmental and economic benefits. On the one hand, it can directly become a raw material or additive for feed production. On the other hand, high-value bioactive metabolites such as saponins and flavonoids can still be extracted from the residue. The emergence of this measure can not only effectively alleviate the environmental pressure caused by large-scale extraction of AOPs in the actual production process but also enable more comprehensive utilization of *A. officinalis*. It can effectively extend the agricultural product industry chain, create new economic growth points, facilitate the comprehensive utilization of resources, and form a circular process from resources to products and renewable resources.

In general, the future research direction of AOPs should focus on deepening the structure–activity relationship studies, promoting the function-oriented precise preparation technology, expanding research on new biological activities, and promoting the development and application of high-yield value-added products and waste resource utilization. These efforts will promote the in-depth development of AOP research from description to mechanism and application, thereby facilitating the value transition.

## 10 Conclusion

*A. officinalis*, as a traditional edible plant, is highly valued by consumers due to its rich nutritional value and unique taste. The structural characteristics of the plant itself are highly compatible with the fermentation technology process. Fermentation technology, as a research hotspot in recent years, has been fully applied in the study of AOPs. It is foreseeable that the changes in chemical structures and biological activities of AOPs before and after fermentation will remain the main research objects in the coming years. At the same time, due to the plant morphology of *A. officinalis* itself, its polysaccharides are mainly composed of cellulose and hemicellulose structures. In addition, *A. officinalis* is relatively inexpensive. Therefore, when studying plant fermentation processes, *A. officinalis* will be a representative research object.

In terms of chemical structure, the complex monosaccharide composition leads to significant heterogeneity in AOPs. It also suggests that it is difficult to determine which structures are responsible for which specific activity. This has also created significant obstacles for the study of structure–activity relationships. Synthesis and modification have not been applied in the structural research of AOPs, making it difficult to obtain uniform polysaccharides with specific sequences. This is also the focus and difficulty researchers will face in subsequent studies. Meanwhile, most existing research mostly focuses on *in vitro* and animal experiments, and clinical translational studies are still lacking. At present, existing research mainly focuses on gut microbiota. On the one hand, this method helps researchers understand the mechanisms of action of AOPs *in vitro* quickly and accurately and *in vivo*. Still, on the other hand, it deprives researchers of more possibilities for studying the mechanisms of AOPs. Therefore, research on AOPs activities should gradually shift from observing phenomena to analyzing mechanisms, along with from single thinking to systematic thinking. In subsequent research, attention should be paid to combining multi-omics and bioinformatics, using isotope labeling and biochemical tools to understand global effects and systematically solve complex problems.

As mentioned above, conducting in-depth research on AOPs as a natural active molecule with broad prospects in functional foods, drug development, and precision medicine is necessary. To better industrialize the research results and strengthen the connection between their foundation and application, this article reviews (Sui et al., 2017; Subramani et al., 2023) the existing literature on AOPs.
